# The role of impaired oxidative base lesion repair in Alzheimer’s disease

**DOI:** 10.1002/alz.090686

**Published:** 2025-01-03

**Authors:** Milena A Egiazarian, Andreas Abentung, Teri S Sakshaug, Emily E Karabeika, Priyanka R Hanumaihgari, Gøril Rolfseng Grøntvedt, Sigrid B Sando, Magnar Bjørås, Katja Scheffler

**Affiliations:** ^1^ Norwegian University of Science and Technology, Trondheim Norway; ^2^ University Hospital of Trondheim, Trondheim Norway; ^3^ University Hospital of Oslo, Oslo Norway

## Abstract

**Background:**

Accumulation of oxidative DNA damage and its inefficient repair is a contributing factor to Alzheimer’s disease (AD), yet the underlying mechanism remains largely unknown. Novel AD mouse models deficient for oxidative DNA damage repair were developed and characterized to better understand their impact on AD progression. In addition, vascularized cerebral organoids from AD patients were generated to translate findings to a human model of AD.

**Method:**

AD mice deficient for oxidative DNA repair enzymes were collected at different ages and amyloid β (Aβ) accumulation quantified by enzyme‐linked immunosorbent assays and immunostaining. Astrocytes and microglia numbers were quantified by immunostaining and inflammatory responses determined by gene expression analyses. DNA damage was measured by mass spectrometry and immunostaining of DNA damage marker. Behavioral analyses were performed to assess cognitive function. Cerebral organoids were fused with vessel organoids generated form AD patients and healthy controls. Vascularization and AD pathology was determined by immunostaining of specific vessel marker and mesoderm‐derived microglia, and Aβ and neuronal marker, respectively.

**Result:**

Aβ accumulation was significantly altered upon loss of oxidative DNA repair in AD mice in an age‐ and sex‐dependent manner. Microglia and astrocyte number changed significantly during disease progression and were either reduced or increased dependent on the oxidative DNA repair enzyme deficiency and sex of the mice. Level of DNA damage in the brain was only moderately increased or not affected. Cognitive function was significantly impaired in AD mice deficient for oxidative DNA repair enzymes. Cerebral organoids fused with vessel organoids developed vascular structures and showed integration of microglia cells. Aβ accumulated specifically in AD patient‐derived organoids compared to healthy controls and showed signs of neurodegeneration.

**Conclusion:**

The repair of oxidative DNA damage is critical for processes associated with AD pathogenesis, in particular neuroinflammation and important to maintain cognitive function. AD patient‐derived vascularized cerebral organoids are immunocompetent and recapitulate AD pathology which will be advantageous to translate findings from mice to humans.

